# Investigation of TNF-α and IL-6 Levels in the Sera of Non-Melanoma Skin Cancer Patients

**DOI:** 10.29252/ibj.25.2.88

**Published:** 2020-09-01

**Authors:** Mehdi Ghahartars, Shabnam Abtahi, Zahra Zeinali, Mohammad Javad Fattahi, Abbas Ghaderi

**Affiliations:** 1 Department of Dermatology, School of Medicine, Shiraz University of Medical Sciences, Shiraz, Iran;; 2 Shiraz Institute for Cancer Research, School of Medicine, Shiraz University of Medical Sciences, Shiraz, Iran,; 3 Department of Immunology, School of Medicine, Shiraz University of Medical Sciences, Shiraz, Iran

**Keywords:** Biomarkers, Cytokines, Interleukin-6, Tumor Necrosis Factor-alpha

## Abstract

**Background:**

TNF-α and IL-6 are both pleiotropic cytokines playing major roles in cancer-associated cytokine networks. They have previously been investigated for their function in skin malignancies, mostly melanomas, and studies on NMSC patients are relatively rare. In this study, we aimed to assess the associations of serum levels of IL-6 and TNF-α with NMSCs and its clinicopathological features.

**Methods:**

This cases-control study was carried out to investigate the serum levels of TNF-α and IL-6 in 70 NMSC patients, in comparison with 30 healthy individuals, by means of flow cytometric bead-based immuneoassay.

**Results:**

Serum levels of both TNF-α and IL-6 were significantly higher in NMSC patients (6.470 vs. 4.355 pg/ml; *p* = 0.0468, respectively), compared to healthy individuals (3.205 vs. 0.000 pg/ml; *p* = 0.0126, respectively). In the subgroup analysis, SCC patients had higher serum levels of IL-6 compared to healthy individuals (3.445 vs. 0.000 pg/ml; *p* = 0.0432). No other significant differences were observed in the serum levels of these two cytokines among different clinicopathological subgroups of the patients.

**Conclusion:**

The increased levels of TNF-α and IL-6 in NMSC patients can be introduced as an epiphenomenon of a complex cancer-induced cytokine cascade.

## INTRODUCTION

Skin cancer, the most common human malignancy, is clinically diagnosed and confirmed by histopathological examination of biopsy specimens. While melanoma is the deadliest skin cancer, the majority of skin neoplasms are NMSCs, including SCC and BCCs^[^^1^^]^. It is well recognized that immune responses are critical defense mechanisms against NMSCs; however, some aspects and biologic effects of these responses might stimulate neoplastic transformation and progression. Chronic inflammation is considered as a major driving force for epidermal cell oncogenesis^[^^2^^]^, and through the production of cytokines, chemokines, and growth factors, immune responses may serve as tumor promoters by supporting tumor cell proliferation, angiogenesis, and inhibiting programmed cell death^[^^3^^]^. 

The use of serum cytokine levels for cancer prognosis is among the well-established methods, and raised serum concentration of pro-inflammatory cytokines such as IL-1, IL-6, IL-8, or TNF-α are usually correlated with tumor prognosis^[^^4^^-^^7^^]^. TNF-α and IL-6 appear to be the major components of cancer-associated cytokine networks, usually resulting in a cytokine-associated inflammation in cancer microenvironment^[^^8^^-^^10^^]^. 

TNF-α is a pleiotropic cytokine that shows a dual role in cancer progression^[^^11^^,^^12^^]^. While living up to its name by inducing cancer cell death, TNF-α is involved in inflammation-associated carcinogenesis by means of supporting tumor cells growth, survival, differentiation, invasion, metastases, and subverting the immune responses^[^^11^^-^^14^^]^. A number of reports have indicated that the serum levels of TNF-α are elevated in various malignancies, with higher levels in preneoplastic and neoplastic tissues^[^^12^^]^. Although showing some anti-inflammatory properties, IL-6 is well known as a pro-inflammatory cytokine exhibiting a similar function to TNF-α-induced inflammation^[^^7^^]^. In the majority of clinical studies on patients with different types of cancer, the IL-6 serum levels increase, possibly reflecting a tumor-type-independent systemic phenomenon. Moreover, it has been suggested that the elevated serum levels of IL-6 are correlated with disease severity and a worse clinical outcome in cancer patients^[^^8^^]^. 

TNF-α and IL-6 have previously been investigated for their functions in skin malignancies, mostly melanomas^[^^10^^,^^15^^]^, and studies on NMSC patients are relatively fewer. In this study, we measured the serum levels of IL-6 and TNF-α in NMSC patients, using flow cytometric bead-based immune-assay, in an attempt to investigate their association with NMSC pathogenesis and clinicopathological parameters. 

## MATERIALS AND METHODS

**Study groups**

The present case-control study was designed to evaluate the serum levels of TNF-α and IL-6 in NMSC patients. Seventy patients with histopathologically confirmed diagnosis of SCC or BCC were recruited from Faghihi Dermatology Clinic affiliated with Shiraz University of Medical Sciences (Shiraz, Iran).

The demographic and clinicopathologic data of the patients were obtained from the hospital files (Table 1). Patients with the history of malignancies or other immunological disorders, and those with metastatic NMSC were excluded from the study. The control group included 30 age-sex-matched healthy individuals from the same geographic region with no history of malignancies or immunological disorders and with no symptoms of infection at the time of sampling. 

**Cytokine measurement**

Venous blood sample (5 ml) was collected from each participant, and the samples were centrifuged for 10 minutes at 2500 ×g. The sera were separated and stored at -70 °C until analysis. The LEGENDplex™ Human Th Cytokine Panel (Cat. No. ‎740722, Biolegend, USA) was used for the measurement of IL-6 and TNF-α serum levels, according to manufacturer’s instructions. In summary, two sets of microbeads, which were coated with capture antibodies specific to IL-6 and TNF-α, were prepared. The different bead populations were mixed and incubated with samples and recombinant standards. Thereafter, PE-conjugated antibodies were added and incubated at room temperature for 1 hour, to form sandwich complexes. After washing, the bead-pellet samples were re-suspended in the wash buffer and analyzed in the FL-3 channel of a BD FACS Calibur flow cytometer (BD Bioscience, USA). Based on the difference in the size and internal fluorescence intensity of beads, analyte-specific populations can be segregated. The results were generated in graphic and tabular format by using the BioLegend’s LEGENDplex^TM^ Data Analysis Software.‎ The assay sensitivities were 2.010 pg/ml for IL-6 and 1.970 pg/ml for TNF-α. 

**Table 1:** Clinicopathologic characteristics of NMSC patients and their respective TNF-α and IL-6 serum levels in each subgroup

**Variables**	**N** ** (%)**	**TNF-α serum level (pg/ml)** ^1^	***p *** ***value*** ^2^	**IL-6 serum level (pg/ml)** ^1^	***p value*** ^2^
Gender					
Male Female	52 (74.28)	7.950 (4.815-16.155)	0.015^*^	2.610 (0.000-6.710)	0.191
	18 (25.72)	4.710 (1.523-8.418)		4.610 (2.018-6.890)	
					
Pathology					
SCC BCC	40 (57.14)	6.700 (4.400-17.863)	0.809	3.445 (0.000-6.983)	0.809
	30 (42.86)	6.365 (3.873-12.353)		2.815 (0.000-6.800)	
					
Tumor Site					
Sun-exposed Not sun-exposed	55 (91.60)	6.830 (4.400-18.340)	0.350	4.250 (2.290-6.450)	0.876
	5 (8.40)	2.810 (2.030-10.820)		3.040 (0.000-6.830)	
					
Number of lesions					
One Multiple	51 (85.00)	6.570 (4.400-18.340)	1.000	4.250 (2.290-6.450)	0.828
	9 (15.00)	6.360 (3.610-12.353)		4.770 (2.018-6.890)	

^ 1^Median (1^st^ -3^rd^ interquartile); ^2^Mann-Whitney-U test; ^*^Statistically significant

**Statistical analysis**

For the statistical analysis, SPSS version 22.0 (IBM Corp., Armonk, NY, USA) was used. The Kolmogorov-Smirnov test was used to analyze the normality of the data. Appropriate parametric (t-test and one-way ANOVA) or non-parametric (Mann-Whitney U and Kruskal-Wallis) tests were performed for comparison between groups. Variables with normal distribution are presented as mean ± SD, otherwise as median and first and third interquartile ranges. Frequencies are presented as percentages. *p *< 0.05 was considered statistically significant.

**Ethical statement**

The above-mentioned sampling protocols were designed in concordance with the Declaration of Helsinki principles^[^^16^^]^, and the Medical Ethics Committee of Shiraz University of Medical Sciences approved the study (IR.sums.med.rec.1397. 164). All participants consented (in a written form) to be involved in this research. 

## RESULTS

In total, 70 newly diagnosed NMSC patients, along with 30 healthy age-sex-matched individuals, as control group, were recruited. None of the patients had lymph node involvement or distant metastasis. The mean age of patients was 68.4 ± 13.9, with 74.3% (n = 52) of them being male. According to histopathologic analysis, patients were subgrouped into those diagnosed as BCC (n=30, 42.9%) and SCC (n=40, 57.1%). Other clinicopathological features of the patients along with their TNF-α and IL-6 serum levels are presented in Table 1. 

**Fig. 1:**
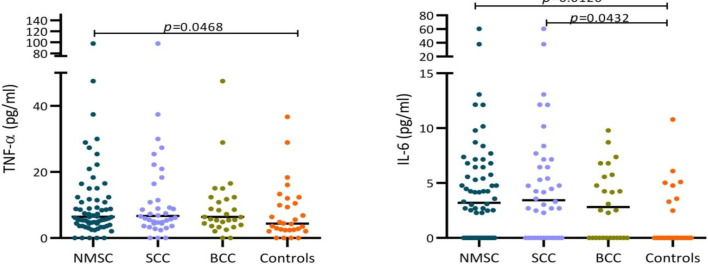
Scatter dot plot diagram of TNF-α and IL-6 serum levels. The middle line represents the median. The ends of the whiskers represent 10-90 percentile.(A) NMSC patients had significantly higher levels of TNF-α in their sera compared to the healthy controls (6.470 vs. 4.355 pg/ml; *p* = 0.0468). (B) IL-6 levels were significantly higher in the NMSC patients than controls (3.205 vs. 0.000 pg/ml; *p* = 0.0126) and in SCC patients than healthy individuals (3.445 vs. 0.000 pg/ml; *p* = 0.0432)

With regard to TNF-α, NMSC patients had significantly higher levels of this cytokine in their sera compared to the healthy controls (Fig. 1A; 6.470 vs. 4.355 pg/ml; *p = *0.0468). Subgroup analysis showed no difference between TNF-α serum levels of SCC and BCC patients (*p > *0.9999). By comparing IL-6 serum concentration in NMSC patients with healthy individuals, we observed that IL-6 levels were significantly higher in patients than the controls (Fig. 1B; 3.205 vs. 0.000 pg/ml; *p = *0.0126). In the subgroup of the patients, according to pathologic diagnosis, the serum levels of IL-6 were not changed between the SCC and BCC patients (*p > *0.9999). However, SCC patients had elevated levels of IL-6 in their sera in comparison to the healthy individuals (Fig. 1B; 3.445 vs. 0.000 pg/ml; *p = *0.0432). Not such difference was found between the BCC patients and controls (*p = *0.1966). There was also no significant difference in the serum levels of these two cytokines among other different clinicopathological subgroups of the patients. 

## DISCUSSION

TNF-α and IL-6 are both pleiotropic cytokines capable of contributing to the immunological defense mechanisms based on their microenvironment. Hence, they can either induce immune-mediated tumor regression or enhance tumor progression and distance metastasis^[^^7^^,^^8^^,^^12^^,^^15^^,^^17^^]^. In this study, we observed that the levels of these two cytokines were significantly higher in NMSC patients than to healthy individuals, but there was no difference in their serum levels among SCC and BCC patients. These results are in consistent with previous studies showing the association of higher TNF-α ad IL-6 serum levels with cancer, independent of the original tumor type^[^^8^^,^^18^^]^. These observations might be interpreted as a paraneoplastic cytokine pattern, which is not related to the tumor pathology^[^^18^^]^. 

TNF-α is a homotrimeric multifunctional cytokine produced primarily by immune cells (mainly monocytes and macrophages), with both local (e.g. tumor microenvironment) and systemic effects^[^^19^^]^. This cytokine has critical roles in immune responses, particularly innate and cellular immunity activation^[^^19^^]^. The anti-tumor activity of TNF-α is now well established and is thought to be mediated through a variety of mechanisms, e.g. induction of cellular apoptosis, T-effector cell activation, and tumor microvasculature collapse^[^^20^^]^. Interestingly, the use of TNF-α inhibitors in autoimmune diseases seems to be associated with the increased risk of NMSCs occurrence, in particular^[^^21^^,^^22^^]^. On the other hand, consistent with our observations in NMSC patients, the increased serum levels of TNF-α has been described in various independent cancer types^[^^18^^]^. These seemingly paradoxical observations can be attributed to the TNF-α axis acting a dual function in tumor progression^[^^23^^]^. TNF-α signaling is transmitted through two cell surface receptors, TNFR-1 and TNFR-2. It is believed that TNFR-1, which is expressed ubiquitously, conveys both tumor inhibition and promotion through pro-apoptotic or pro-survival signals on cancer and immune cells. However, TNFR-2, which its expression is restricted to neoplastic cells and suppressive immune cells, appears to have mainly a tumor-promoting effect by accelerating tumor growth, regulating the survival and function of several types of immunosuppressive cells*,* and promoting angiogenesis by inducing IL-6 secretion^[^^23^^,^^24^^]^. IL-6 is up-regulated in inflammatory processes such as infections, trauma, autoimmune diseases, and cancer^[^^15^^]^. Serum concentrations of IL-6 have been reported to be increased in diverse types of cancers and seem to be associated with tumor progression and prognosis^[^^8^^,^^18^^]^. It has been reported that different cell types in the tumor niches produce IL-6, and the IL-6/JAK/STAT3 pathway is aberrantly hyperactivated in many cancers, leading to the increased tumor-cell growth and progression, survival, and metastasis. In this respect, inhibitors of IL-6 have received U.S. Food and Drug Administration approval for the treatment of various malignancies^[^^25^^]^.  

In a model for human SCC, designed by Lederle and colleagues^[^^9^^]^, it has been revealed that IL-6 play a pivotal role in mediating neoplastic transformation from a benign to an invasive tumor phenotype. The same group have also shown that IL-6 may act to trigger a complex of cytokine network that includes VEGF, GM-CSF, IL-8, MCP-1, and MMP-1, the mediators and cytokines involved in the promotion of malignant progression by autocrine and paracrine mechanisms^[^^9^^,^^26^^]^.

The increased levels of TNF-α and IL-6 may be a reflection of a cascade of inflammatory response either as primary or secondary contributing factors in the pathogenesis of patients with NMSC. Studies in larger scales and *in vitro* and *in vivo* models of skin cancer might further clarify the causality of these associations. 

## References

[1] Esteva A, Kuprel B, Novoa RA, Ko J, Swetter SM, Blau HM, Thrun S (2017). Dermatologist-level classification of skin cancer with deep neural networks. Nature.

[2] Rangwala S, Tsai KY (2011). Roles of the immune system in skin cancer. Br j dermatol.

[3] Grivennikov SI, Greten FR, Karin M (2010). Immunity inflammation and cancer. Cell.

[4] Lin WW, Karin M (2007). A cytokine-mediated link between innate immunity, inflammation, and cancer. The journal of clinical investigation.

[5] Lewis AM, Varghese S, Xu H, Alexander HR (2006). Interleukin-1 and cancer progression: the emerging role of interleukin-1 receptor antagonist as a novel therapeutic agent in cancer treatment. Journal of translational medicine.

[6] Balkwill F (2006). TNF-alpha in promotion and progression of cancer. Cancer metastasis reviews.

[7] Mauer J, Denson JL, Brüning JC (2015). Versatile functions for IL-6 in metabolism and cancer. Trends in immunology.

[8] Lippitz BE, Harris RA (2016). Cytokine patterns in cancer patients: A review of the correlation between interleukin 6 and prognosis. Oncoimmunology.

[9] Lederle W, Depner S, Schnur S, Obermueller E, Catone N, Just A, Fusenig NE, Mueller MM (2011). IL-6 promotes malignant growth of skin SCCs by regulating a network of autocrine and paracrine cytokines. International journal of cancer.

[10] Donia M, Kjeldsen JW, Svane IM (2016). The controversial role of TNF in melanoma. Oncoimmunology.

[11] Balkwill F (2009). Tumour necrosis factor and cancer. Nature reviews cancer.

[12] Wang X, Lin Y (2008). Tumor necrosis factor and cancer buddies or foes?. Acta pharmacologica Sinica.

[13] Kalliolias GD, Ivashkiv LB (2016). TNF biology, pathogenic mechanisms and emerging therapeutic strategies. Nature reviews rheumatology.

[14] Sasi SP, Yan X, Enderling H, Park D, Gilbert HY, Curry C, Coleman C, Hlatky L, Qin G, Kishore R, Goukassian DA (2012). Breaking the 'harmony' of TNF-α signaling for cancer treatment. Oncogene.

[15] Bridge JA, Lee JC, Daud A, Wells JW, Bluestone JA (2018). Cytokines chemokines, and other biomarkers of response for checkpoint inhibitor therapy in skin cancer. Frontiers in medicine (Lausanne).

[16] World Medical Association (2013). World Medical Association Declaration of Helsinki: ethical principles for medical research involving human subjects. JAMA.

[17] Wajant H (2009). The role of TNF in cancer. Results and problems in cell differentiation.

[18] Lippitz BE (2013). Cytokine patterns in patients with cancer: a systematic review. The lancet oncology.

[19] Holbrook J, Lara-Reyna S, Jarosz-Griffiths H, McDermott M (2019). Tumour necrosis factor signalling in health and disease. F1000 research.

[20] Josephs SF, Ichim TE, Prince SM, Kesari S, Marincola FM, Escobedo AR, Jafri A (2018). Unleashing endogenous TNF-alpha as a cancer immunotherapeutic. Journal of translational medicine.

[21] Jain A, Singh JA (2013). Harms of TNF inhibitors in rheumatic diseases: a focused review of the literature. Immunotherapy.

[22] Chen Y, Friedman M, Liu G, Deodhar A, Chu CQ (2018). Do tumor necrosis factor inhibitors increase cancer risk in patients with chronic immune-mediated inflammatory disorders?. Cytokine.

[23] Ham B, Fernandez MC, D'Costa Z, Brodt P (2016). The diverse roles of the TNF axis in cancer progression and metastasis. Trends in cancer research.

[24] Sheng Y, Li F, Qin Z (2018). TNF Receptor 2 makes tumor necrosis factor a friend of tumors. Frontiers in immunology.

[25] Johnson DE, O'Keefe RA, Grandis JR (2018). Targeting the IL-6/JAK/STAT3 signalling axis in cancer. Nature reviews clinical oncology.

[26] Depner S, Lederle W, Gutschalk C, Linde N, Zajonz A, Mueller MM (2014). Cell type specific interleukin-6 induced responses in tumor keratinocytes and stromal fibroblasts are essential for invasive growth. International journal of cancer.

